# Limiting Premenstrual Endometrial Hypoxia Inducible Factor 2 Alpha May Fine-Tune Endometrial Function at Menstruation

**DOI:** 10.1210/clinem/dgae630

**Published:** 2024-09-11

**Authors:** Rocío Martínez-Aguilar, Bethan M Rowley, Catherine Walker, Hilary O D Critchley, Peter Carmeliet, Jacqueline A Maybin

**Affiliations:** Centre for Reproductive Health, Institute for Regeneration and Repair, University of Edinburgh, Edinburgh, EH16 4UU, UK; Centre for Reproductive Health, Institute for Regeneration and Repair, University of Edinburgh, Edinburgh, EH16 4UU, UK; Centre for Reproductive Health, Institute for Regeneration and Repair, University of Edinburgh, Edinburgh, EH16 4UU, UK; Centre for Reproductive Health, Institute for Regeneration and Repair, University of Edinburgh, Edinburgh, EH16 4UU, UK; Laboratory of Angiogenesis and Vascular Metabolism, VIB-KU Leuven Center for Cancer Biology, Leuven, 3000, Belgium; Center for Biotechnology, Khalifa University of Science and Technology, Abu Dhabi, CF9X+CP9, United Arab Emirates; Centre for Reproductive Health, Institute for Regeneration and Repair, University of Edinburgh, Edinburgh, EH16 4UU, UK

**Keywords:** heavy menstrual bleeding, endometrium, hypoxic, progesterone, HIF, repair

## Abstract

**Context:**

Heavy menstrual bleeding (HMB) is common and debilitating, but the precise endometrial mechanisms causing increased menstrual blood loss (MBL) remain undefined. We have previously identified a role for hypoxia in endometrial repair following progesterone withdrawal.

**Objective:**

As hypoxia inducible factor 2 alpha (HIF2A) is known to alter vascular function in other tissues, we hypothezised that endometrial HIF2A is involved in premenstrual optimization of endometrial function during the secretory phase to limit MBL.

**Results:**

Women with objective HMB had higher endometrial HIF2A during the mid-secretory phase when compared to those with normal MBL (*P* = 0.0269). In a mouse model of simulated menses, genetic or pharmacological manipulation of HIF2A did not significantly affect endometrial breakdown/repair, volume of MBL or endometrial hypoxia. However, 88% of *Hif2a* heterozygote mice reached early-full repair by 24 hours vs only 65% of wild-type mice. Mean MBL was 0.39 μL (±0.67) in *Hif2a* heterozygote mice vs 0.98 μL (±0.79) in wild-type mice. Conversely, when we increased HIF2A before menstruation, 11% reached early repair by 8 hours vs 30% of vehicle-treated mice. Mean MBL was 2.61 μL (±1.10) in mice with HIF2A stabilization and 2.24 μL (±1.14) in vehicle-treated mice. These nonsignificant but consistent trends indicate that increased endometrial HIF2A may contribute to delayed endometrial repair and HMB.

**Conclusions:**

Increased HIF2A in the secretory endometrium is unlikely to be sufficient to account for the phenotype of HMB, but limitation of HIF2 levels may optimize endometrial function at menstruation.

Heavy menstrual bleeding (HMB) is a common reason for gynecological consultations (1), with an estimated prevalence of 30% among European women of reproductive age (2) and a range of 10% to 35% among US women (3). HMB has a detrimental effect on the quality of life of those affected and imposes a significant socioeconomic burden on society as a whole (4). Current treatments heavily rely on hormonal preparations (eg, combined oral contraceptive pill, levonorgestrel intrauterine system), which, although they can be effective, often result in intolerable side effects and act as contraceptives/reduce the chances of conception during their use (5). Due to treatment failure or side effects, up to 33% of affected individuals resort to hysterectomy (surgical removal of the uterus) (5, 6). Underresearch of women's health (7), particularly endometrial physiology and the specific underlying endometrial mechanisms that cause HMB, hinders progress in the development of non-hormonal, targeted, and effective treatments for this condition.

The endometrium is a dynamic tissue highly responsive to environmental cues to initiate cyclical remodeling and regeneration (8). In particular, hormonal stimuli trigger complex signaling pathways to ensure adequate endometrial adaptation, including cellular differentiation and vascular maturation during decidualization, to accommodate pregnancy or swiftly return to an unprimed stage (9). When fertilization does not occur, a decrease in progesterone (P4) and estradiol (E2) levels initiates the cascade of events responsible for endometrial breakdown, menstrual bleeding, and subsequent repair (9). Normal endometrial repair requires (1) a prompt resolution of the inflammatory processes that culminate in endometrial tissue destruction, (2) vasoconstriction of specialized blood vessels to limit menstrual flow, (3) efficient hemostasis, and (4) scarless reepithelialization of the denuded surface. Aberrations in these processes potentially lead to the symptom of HMB, but more in-depth knowledge of the cellular and molecular events that limit menstrual blood loss (MBL) are needed.

The concept that hypoxia plays an important role during menstruation has been debated since the 1940s (10-16). More recently, human in vivo data (17) and mechanistic mouse studies (18) revealed that hypoxia is present in the menstrual endometrium and drives endometrial repair following shedding to limit MBL. The oxygen-sensing protein hypoxia-inducible factor (HIF) is the main transcription factor that controls the cellular response to hypoxia (19). In the presence of oxygen, its alpha subunit is hydroxylated by prolyl hydroxylase enzymes and tagged for rapid proteasomal degradation. In hypoxic conditions, prolyl hydroxylase enzymes become inactive, allowing translocation of the alpha subunit to the nucleus where it can dimerize with the beta subunit. This alpha/beta complex can then bind to hypoxia-response elements to enhance transcription of genes involved in angiogenesis, energy metabolism, and tissue remodeling. The HIF alpha subunit has 2 main isoforms, HIF1A and HIF2A, with distinct but overlapping gene specificities (20).

Fluctuations in HIF protein levels across the cycle have been previously described (18), with HIF1A found to be maximal during the menstrual phase, while HIF2A was exclusively present during the early-mid secretory phase (18). In those with objectively measured HMB (blood loss > 80 mL/cycle) (5), menstrual levels of HIF1A have been shown to be lower compared to those with normal menstrual bleeding (NMB) (18). Differences in HIF2 in those with objective HMB and NMB have not previously been reported. Mechanistic studies on mouse models revealed that lower HIF1A delayed endometrial repair (18). Whereas the role of HIF1A in the endometrial context of HMB is starting to be elucidated (10, 18), the regulation and role of HIF2A in the nonpregnant endometrium is yet to be studied. Mutations in *EPAS1* (encoding human HIF2A) have a protective effect on high-altitude-associated intrauterine growth restriction and reproductive loss (21, 22), and HIF2A deficiency in a mouse implantation model revealed a key role in endometrial receptivity, embryonic implantation, and survival (23). Moreover, HIF2A is critical in pulmonary and cardiovascular remodeling events (24, 25). Given that HIF2 regulates transcription of a host of downstream factors involved in angiogenesis, apoptosis, metabolism, and cell proliferation (26, 27), we wished to determine its presence and role in the nonpregnant endometrium, where such processes are fundamental for normal function. Based on this evidence, we hypothesized that limitation of endometrial HIF2A levels is required for optimal premenstrual conditioning of endometrial vasculature to facilitate subsequent menstrual vasoconstriction, leading to the local tissue hypoxia that triggers endometrial repair and limits MBL.

## Methods

### Human Tissue Collection

Written informed consent was obtained from participants and ethical approval granted from Lothian Research Ethics Committee (REC 07/S1103/29, 20/ES/0119). Endometrial biopsies (n = 29) were collected with a suction curette (Pipelle®, Laboratorie CCD, Paris, France) from women (median age 42 years, range 26-50) attending gynecological outpatient departments in NHS Lothian. Uterine samples comprising endometrial and myometrial layers (full-thickness endometrial samples, n = 5; 2 representative depicted) from women undergoing hysterectomy for noncancerous conditions (eg, abnormal uterine bleeding) in NHS Lothian were also collected and fixed in 4% neutral-buffered formalin for immunohistochemical studies only.

Regarding inclusion criteria, all participants reported regular menstrual cycles (21-35 days) and no exogenous hormone exposure for 2 months prior to biopsy. Exclusion criteria included large fibroids (>3 cm), known endometriosis, or reproductive tract cancer. Endometrial biopsies were collected, divided, and immediately (1) placed in RNA later solution (Ambion, Warrington, UK), (2) snap-frozen for future protein Western blot analysis and (3) placed in NBF. They were categorized as mid secretory based on consistency across 3 parameters (1) histological dating using criteria of Noyes et al (28), (2) reported last menstrual period, and (3) serum P4 and E2 concentrations at the time of biopsy.

MBL was objectively measured using a modified alkaline hematin method (29). HMB was defined as a blood loss of >80 mL per cycle (5), resulting in n = 5 with HMB and n = 4 with NMB (<80 mL per cycle). Participants were given the same brand of menstrual products (Tampax® tampons/Always® towels, Procter & Gamble, Cincinnati, OH, USA) with verbal and written instructions on collection. The technique was previously validated in our laboratory using a known volume of whole blood applied to menstrual products.

### Mouse Studies

All experimental animal procedures were approved by the University of Edinburgh ethical committee and performed in accordance with the Animals Scientific Procedures Act (1986) of the UK Home Office (PPL 70/8754 and PP4688615). Experimental request approval was obtained by named veterinary surgeons from the University of Edinburgh for every protocol involving rodent work.

Female C57BL/6JOlaHsd mice were purchased from Envigo RMS (Loughborough, UK). *Hif2a* heterozygous mice (*Hif2a^+/−^*, mixed-background 129/Sv × CD-1) and *Hif1a* heterozygous mice (*Hif1a^+/−^* mice, mixed-background 129/Sv × CD-1) were kindly donated by Professor P. Carmeliet (24, 30). Littermate female controls [*Hif-1a*^+/+^ or *Hif-2a*^+/+^, hereafter referred to as wild-type (WT)] were mated with *Hif1a*^+/−^ or *Hif2a*^+/−^ males, respectively, and offspring genotyped. For the *Hif2a* subfertility analysis, both WT and *Hif2a*^+/−^ females were mated with male WT littermate controls.

### Simulated Mouse Model of Menstruation

**Figure 1.**
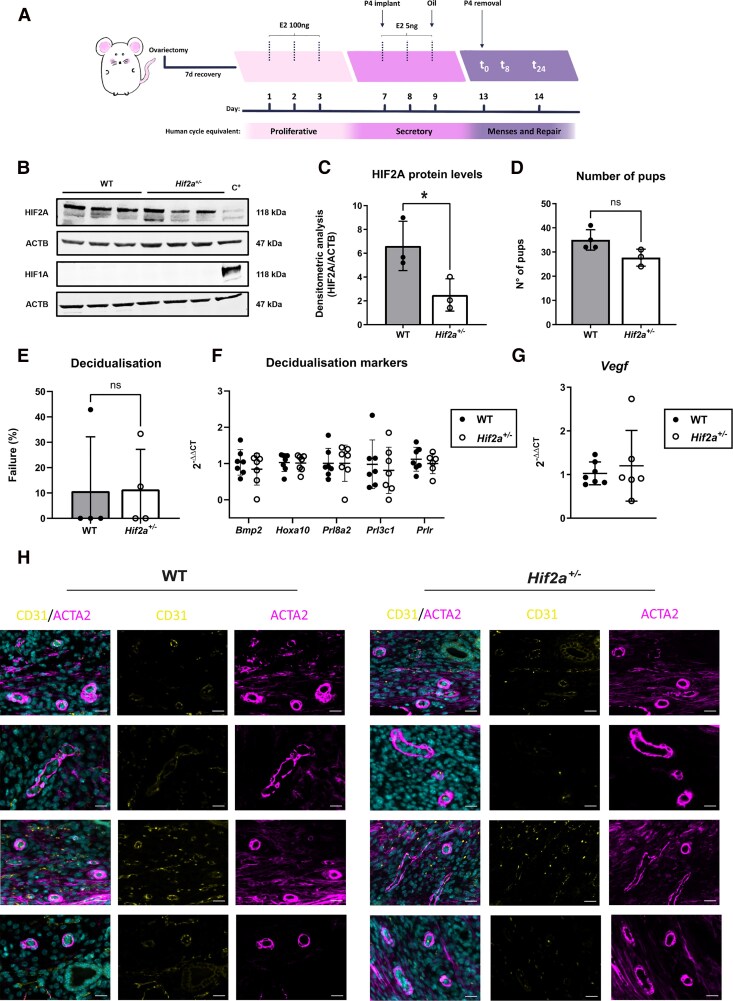
*Hif2a^+/−^* mice do not show subfertility, compromised decidualization, or vascular alterations at t_0_ compared to WT in our mouse model of simulated menses. (A) Mouse model of simulated menses. E2 administration starts 7 days after ovariectomy surgery. (B) Confirmation of a reduction in HIF2A protein in *Hif2a*^+/−^ vs WT mice measured by Western blot in uterine tissue prior to P4 withdrawal (t_0_). This was not accompanied by a compensatory increase in HIF1A. Protein extracts from the murine endothelial cell line 3B-11 subjected to hypoxia for 8 hours were used as a positive control (C^+^). (C) Densitometry of uterine protein extracts for HIF2A from WT vs *Hif2a*^+/−^ mice prior to P4 withdrawal (t_0_). n = 3 per experimental group. *P <.05, unpaired *t*-test. (D) Total number of pups in WT vs *Hif2a^+/−^* female breeders during a 3-month breeding trial. Each individual datapoint represents a different breeding pair. (E) Percentage of WT and *Hif2a*^+/−^ mice that failed to undergo decidualization (complete absence of uterine horn thickening) in 4 different experimental runs. (F) mRNA expression levels of key decidualization markers (*Bmp2, Hoxa10, Prl8a2, Prl3c1, Prlr*) in uterine tissue from WT vs *Hif2a*^+/−^ mice at the time of decidualization (t_0_). (G) mRNA expression levels of *Vegf* in uterine tissue from WT vs *Hif2a*^+/−^ mice at t_0_. (H) *Hif2a* partial genetic deficiency does not have a significant impact on the coexpression of vessel maturity markers CD31/ACTA2. Uterine endothelial (CD31) and mural cells (ACTA2) staining in WT and *Hif2a^+/−^* mice at the time of decidualization (t_0_). 4′,6-Diamidino-2-phenylindole used as nuclei costain. Scale bar 20 μm. Data represented as mean ± SD.

Abbreviations: ACTB, actin; ACTA2, alpha smooth muscle actin; E2, estradiol; HIF2A, hypoxia inducible factor 2 alpha; P4, progesterone; t_0_: time of progesterone implant removal; t_8_, 8 hours following P4 withdrawal; t_24_, 24 hours following P4 withdrawal; WT, wild-type.

Endometrial breakdown and repair were induced in mice as previously described (18, 31). Briefly, 6- to 9-week-old female mice were ovariectomized to deplete endogenous steroid hormone release and production (Fig. 1A). After a 7-day recovery period, mice received daily subcutaneous (sc) injections of β-E2 in sesame oil (100 ng/100 µL) on days 1 to 3. On day 7, a P4 implant was placed subcutaneously. Mice also received daily sc injections of E2 (5 ng/100 µL) from day 7 to 9. To trigger decidualization, 20 μL of peanut oil was intracervically administered using a nonsurgical embryo transfer device (mNSET™, ParaTechs Corporation, Lexington, KY, USA). P4 withdrawal was induced 4 days after decidualization (day 13) by removal of the sc implant. Mice received an intraperitoneal injection of pimonidazole (60 mg/kg; Hypoxyprobe, Burlington, MA, USA) 1.5 hours prior to culling. Mice were culled by cervical dislocation at the time of P4 withdrawal (t_0_) and 8 hours (t_8_) or 24 hours (t_24_) after P4 withdrawal. Uterine horns were dissected and placed in RNA later, 4% neutral buffered formalin for paraffin embedding or snap-frozen in liquid nitrogen for protein extraction. Any animal with failed decidualization, defined as complete absence of uterine horn thickening, was excluded from the study but noted for decidualization statistics. The percentage of decidualization failure was calculated as the number of mice with complete absence of uterine thickening divided by the total number of mice used in each experimental group.

### Pharmacological Manipulation of HIF2A

**Figure 2.**
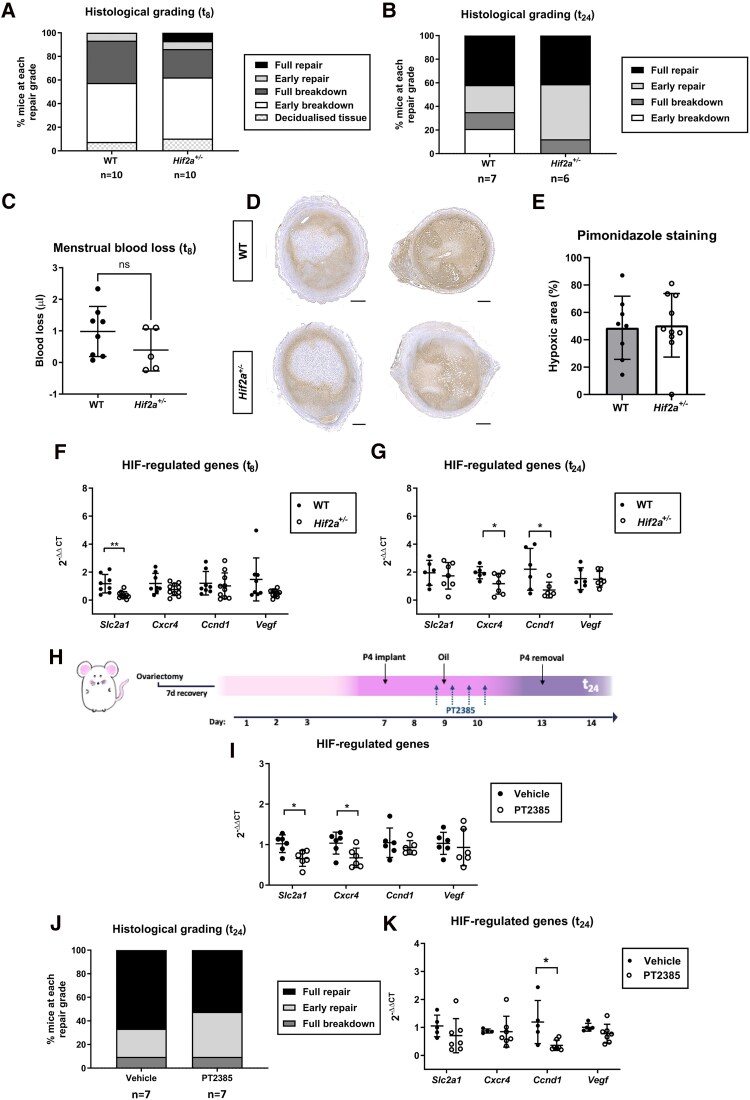
Genetic or pharmacological inhibition of HIF2A does not significantly affect endometrial breakdown or repair. (A) Endometrial histological breakdown/repair score from WT vs *Hif2a*^+/−^ mice at t_8_ (bleeding timepoint). The graph represents the percentage of mice at each histological grade per experimental group. (B) Endometrial histological breakdown/repair score at t_24_ (repair timepoint) in WT vs *Hif2a*^+/−^ mice. (C) Quantification of uterine blood loss in WT vs *Hif2a*^+/−^ mice at t_8_. (D) Representative endometrial pimonidazole staining and (E) quantification of the hypoxic area from WT vs *Hif2a*^+/−^ mice at t_8_. Scale bar 500 μm. (F) mRNA concentrations of hypoxia/HIF downstream targets (*Slc2a1, Cxcr4, Ccnd1, Vegf*) in uterine tissue from WT vs *Hif2a*^+/−^ mice at t_8_. **P <.01, unpaired *t*-test. (G) mRNA concentrations of hypoxia/HIF downstream targets in uterine tissue from WT vs *Hif2a*^+/−^ mice at t_24_. *P <.05, unpaired *t*-test. (H) Pharmacological strategy to selectively inhibit HIF2 during decidualization. Female C57BL/6JOlaHsd mice underwent the mouse model of simulated menses and were treated with the HIF2A selective inhibitor PT2385. On day 9, PT2385 was administered via oral gavage (10 mg/kg) with a total of 4 doses spaced in 12-hour intervals (7 hours prior to the decidualization stimulus, 5 hours, 17 hours, and 29 hours postdecidualization). (I) Significant downregulation of HIF binding targets *Slc2a1* and *Cxcr4* in mouse uterine tissue 4 hours after a single administration of the HIF2A selective inhibitor PT2385. *P <.05, unpaired *t*-test. (J) Endometrial histological breakdown/repair score at t_24_ in C57BL/6J-OlaHsd mice treated with vehicle or the HIF2A inhibitor PT2385. (K) mRNA expression of hypoxia/HIF downstream targets in uterine tissue from vehicle vs PT2385-treated mice at t_24_. *P <.05, unpaired *t*-test. Data represented as mean ± SD, except histological grading graphs (mean only).

Abbreviations: HIF2A, hypoxia inducible factor 2 alpha; P4, progesterone; t_8_, 8 hours following P4 withdrawal; t_24_, 24 hours following P4 withdrawal; WT, wild-type.

To pharmacologically inhibit HIF2 at the time of decidualization, the HIF2 antagonist PT2385 (Selleckchem, Houston, TX, USA) was used. C57BL/6JOlaHsd mice underwent the simulated menstruation protocol described earlier. On day 9, PT2385 was administered via oral gavage (10 mg/kg) with a total of 4 doses spaced in 12-hour intervals (Fig. 2H). The PT2385 solubilization reagents (10% absolute ethanol, 30% polyethylene glycol 400%, and 60% phosphate-buffered saline containing 0.5% methylcellulose and 0.5% Tween 80) were used as vehicle control.

**Figure 3.**
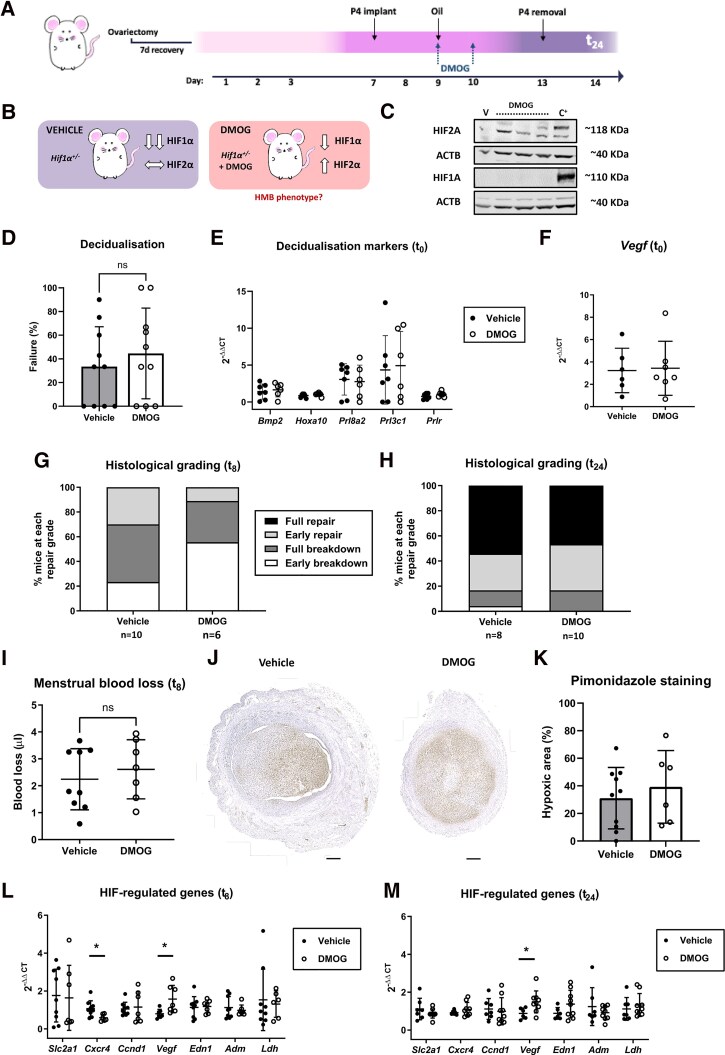
Pharmacological stabilization of HIF2A at the time of decidualization has no significant impact on endometrial breakdown or repair. (A) Pharmacological strategy to selectively increase HIF2 during decidualization. *Hif1a*^+/−^ mice were treated on day 9 (when decidualization is triggered) and 10 with 8 mg/day of the pan-HIF stabilizer DMOG intraperitoneally. Through this approach, (B) pharmacological HIF inhibition will primarily target HIF2A as *Hif1a*^+/−^ mice exhibit a constitutive decrease of HIF1A levels. (C) Protein levels of uterine HIF1A and HIF2A 8 hours after the final administration of DMOG compared to vehicle control (V). Protein extracts from the murine endothelial cell line 3B-11 subjected to hypoxia for 8 hours was used as a positive control (C^+^). (D) Percentage of *Hif1a*^+/−^ mice treated with vehicle vs the HIF stabilizer DMOG that fail to undergo decidualization (complete absence of uterine horn thickening) in 10 different experimental runs. (E) mRNA expression of key decidualization markers (*Bmp2, Hoxa10, Prl8a2, Prl3c1, Prlr*) in uterine tissue from vehicle vs DMOG-treated *Hif1a*^+/−^ mice at t_0_. (F) mRNA concentration of *Vegf* in uterine tissue from vehicle vs DMOG-treated *Hif1a*^+/−^ mice at t_0_. (G) Endometrial histological breakdown/repair score in *Hif1a*^+/−^ mice treated with vehicle vs DMOG at t_0_. Graphs represent percentage of mice at each histological grade per experimental group at t_8_. (H) Endometrial histological breakdown/repair score at t_24_ in *Hif1a*^+/−^ mice treated with vehicle vs DMOG at t_0_. (I) Quantification of uterine blood loss in vehicle vs DMOG-treated *Hif1a*^+/−^ mice at t_8_. (J) Representative endometrial pimonidazole staining and (K) quantification of the hypoxic area in *Hif1a*^+/−^ mice treated with vehicle or DMOG. The analysis timepoint represented is t_8_. Scale bar 500 μm. (L) mRNA expression of HIF downstream targets (*Slc2a1, Cxcr4, Ccnd1, Vegf, Edn1, Adm, Ldh*) in uterine tissue from vehicle or DMOG-treated *Hif1a*^+/−^ mice at t_8_. *P <.05, unpaired *t*-test. (M) mRNA concentration of HIF downstream targets in uterine tissue from vehicle or DMOG-treated *Hif1a*^+/−^ mice at t_24_. *P <.05, unpaired *t*-test. Data represented as mean ± SD, except histological grading graphs (mean only).

Abbreviations: ACTB, actin; DMOG, dimethyloxalylglycine; HIF2A, hypoxia inducible factor 2 alpha; HMB, heavy menstrual bleeding; P4, progesterone; t_0_: time of decidualization; t_8_, 8 hours following P4 withdrawal; t_24_, 24 hours following P4 withdrawal.

Since specific HIF2 stabilizers are not yet available, the prolyl-4-hydroxylase inhibitor dimethyloxaloylglycine (DMOG; Enzo Biochem, Farmingdale, NY, USA) was employed to upregulate general HIF activity at decidualization (P4-dominant phase). However, to selectively increase HIF2A during decidualization, *Hif1a^+/−^* mice underwent the simulated menstruation protocol and were treated on day 9 (at decidualization stimulus) and 10 with 8 mg of DMOG (or PBS) intraperitoneally to mimic the mid-secretory increase in HIF2 detected in women with HMB (Fig. 3A and 3B).

### Objective Measurement of MBL (Mouse)

To measure murine MBL, an adapted version of the human alkaline hematin method (29) was performed. At the time of P4 pellet removal, 1 to 3 cotton pellets were inserted vaginally and stitched in place to collect “menstrual blood”. During the cull, venous blood was also collected by cardiac puncture under terminal anesthesia. A standard curve was created by soaking unused cotton pellets with a range of blood volumes (0.5-35μL). All the cotton pellets were then placed in 5% NaOH for 24 hours prior to squeezing them out and measuring the solution's absorbance at 540 nm on a plate reader (CLARIOstar Plus, BMG Labtech, Ortenberg, Germany). Measurements were adjusted based on the number of uterine horns decidualized per mouse. Anomalies in absorbance values were excluded from analysis if considered outliers after statistical interrogation (n = 1; 2 in WT vs *Hif2a ^+/−^* MBL experiment).

### Histological Analysis

#### HIF2A staining (human endometrium)

Full-thickness human uterine sections (endometrium and myometrium, 5 µm thickness) confirmed as mid-secretory (based on day of cycle, serum ovarian hormone levels, and histological dating) were stained for HIF2A (R and D Systems Cat# AF2997, RRID:AB_2098218) using a 3,3'-diaminobenzidine (DAB) substrate horseradish peroxidase (HRP) kit (Vector Laboratories Cat# SK-4105, RRID:AB_2336520). Single HRP staining and matched isotypes were used as negative controls.

#### Hematoxylin and eosin and immunohistochemistry staining (mouse sections)

Paraffin-embedded mouse uterine sections (5 µm thickness) were stained with hematoxylin and eosin and stage of breakdown/repair was graded by 3 masked independent observers using a previously published scoring system (18) [Supplementary Fig. SA (32)]. Tissue hypoxia was detected using antipimonidazole antibody as per the manufacturer's instructions (hypoxyprobe Cat# HP1-200, RRID:AB_2811309). Briefly, after incubation with the hypoxyprobe kit (1:200), sections were incubated with a mouse-on-mouse HRP polymer detector (Abcam Cat# ab269452, RRID:AB_3351667) followed by a DAB substrate HRP kit incubation. Image analysis was performed by a researcher masked to the experimental groups using the open source software QuPath (33) (v0.4.3). The percentage of endometrial hypoxia was calculated as the hypoxic area/total area of the endometrium. Both regions were manually selected using the *polygon* and *brush* tools in QuPath.

#### Immunofluorescence staining (mouse sections)

Mouse uterine tissue sections were also stained for CD31 (1:100, Abcam Cat# ab28364, RRID:AB_726362) incubated with an AF488 (1:500, Thermo Fisher Scientific Cat# A-11034, RRID:AB_2576217), and alpha smooth muscle actin (1:500, Sigma-Aldrich Cat# C6198, RRID:AB_476856). Single AF488 staining and matched isotypes were used as negative controls. 4′,6-Diamidino-2-phenylindole was used as a nuclei counterstain.

#### Image acquisition

Both mouse and human brightfield/fluorescent images were acquired using the Axioscan Slide Scanner (Zeiss, Oberkochen, Germany) after the creation of tailored hematoxylin and eosin, DAB, and fluorescence staining scan profiles. The selection and cropping of regions of interest was performed using the ZEN software (ZEN 3.4, Zeiss).

### Western Blots

For human endometrial tissue, nuclear protein extracts (10 μg) were obtained using the nuclear extract kit (Active Motif, Carlsbad, CA, USA) following the manufacturer's protocol. Protein quantification was performed using the Bradford assay. Extracts were resuspended in NuPAGE™ LDS Sample Buffer (2X), denatured at 95°C for 5 minutes, and separated on 4% to 12% NuPAGE Bis–Tris Gels (all Thermo Fisher Scientific, Waltham, MA, USA) before wet transfer onto polyvinylidene fluoride membranes (Merck Millipore, Burlington, MA, USA). Membranes were then blocked overnight in a solution containing 5% milk powder in Tris-buffered saline and Tween 20 (0.001%) (TBST). After a 1-hour incubation with primary antibodies (HIF2A, 1:1000, Novus Cat# NB100-132, RRID:AB_10000898; ACTB, 1:5000, Abcam Cat# ab8227, RRID:AB_2305186), membranes were washed with TBST and incubated with the appropriate horseradish peroxidase-conjugated secondary antibodies (1:10.000) before detection using chemiluminescent horseradish peroxidase substrate (Immobilon ECL Ultra Western HRP Substrate, Merck Millipore).

For mouse uterine tissue, nuclear protein extracts (40 μg) were obtained using the nuclear extract kit (Active Motif) following the manufacturer's protocol. Protein quantification was performed using the Pierce™ BCA Protein Assay kit (Thermo Fisher Scientific). Extracts were resuspended in NuPAGE™ LDS Sample Buffer (4X) and NuPAGE™ Sample Reducing Agent (10X) and denatured at 70°C for 10 minutes before loading them in 4% to 12% NuPAGE Bis–Tris Gels (all Thermo Fisher Scientific). The wet transfer was conducted using Immobilon®-FL polyvinylidene fluoride membranes (Merck Millipore). Membranes were then blocked in Odyssey PBS blocking buffer (LI-COR Biosciences, Lincoln, NE, USA) for 90 minutes prior to overnight incubation with primary antibodies (HIF2A, 1:500, R and D Systems Cat# AF2997, RRID:AB_2098218; HIF1A, 1:400, R and D Systems Cat# AF1935, RRID:AB_355064; ACTB, 1:5000, Abcam Cat# ab8227, RRID:AB_2305186). HIF2A possesses multiple bands (120, 118, 83 KDa) as a result of posttranslational modifications. Any of them can be quantified to assess global HIF2 presence. After washing with TBST, membranes were incubated with the appropriate IRDye® secondary antibodies (LI-COR Biosciences) for 1 hour and visualized using the LiCor Odyssey Fc imaging system (LI-COR Biosciences).

### Quantitative Real-Time PCR

Total RNA from whole human endometrial biopsies and mouse uterine tissue were extracted using the RNeasy Mini Kit (QIAGEN, Hilden, Germany) according to the manufacturer's instructions. RNA samples were retrotranscribed using the iScript™ cDNA Synthesis Kit (Bio-Rad, Hercules, CA, USA).

Messenger RNA transcripts were quantified relative to appropriate reference genes (human samples: *18S* and *ATP5B*, mouse samples: *Actb* and *Rpl13*), as determined by geNorm assay (Primerdesign Ltd., Southampton, UK). For *HIF2A* and HIF-regulated genes, specific primers were designed using the universal probe library assay design center and checked with UCSC In-Silico PCR Genome Browser. For decidualization markers, customized TaqMan™ Gene Expression Assays were acquired [Supplementary Table S(32)]. Reactions were performed in triplicate using ABI Quantstudio 5 system (Thermo Fisher Scientific) under standard conditions with TaqPath™ ProAmp™ Master Mix (Thermo Fisher Scientific). Relative quantification was performed using the 2^−ΔΔCt^ method after normalization against controls (human samples: liver cDNA, mouse samples: experimental controls; eg, WT or vehicle mice accordingly).

### Statistical Analysis

Analysis was carried out using GraphPad Prism software (v10.0.2, San Diego, CA, USA). Data was subjected to normality tests and outlier identification using the ROUT method. Exclusion of outliers was restricted to concerns in sample processing and not related to intrinsic interindividual variation. Analysis of 2 groups used unpaired *t* tests where the distribution of data was normal and Mann–Whitney test where data were not normally distributed. A value of *P* <.05 was considered statistically significant.

## Results

### Mid-secretory Endometrial HIF2A Was Increased in Women With HMB

**Figure 4.**
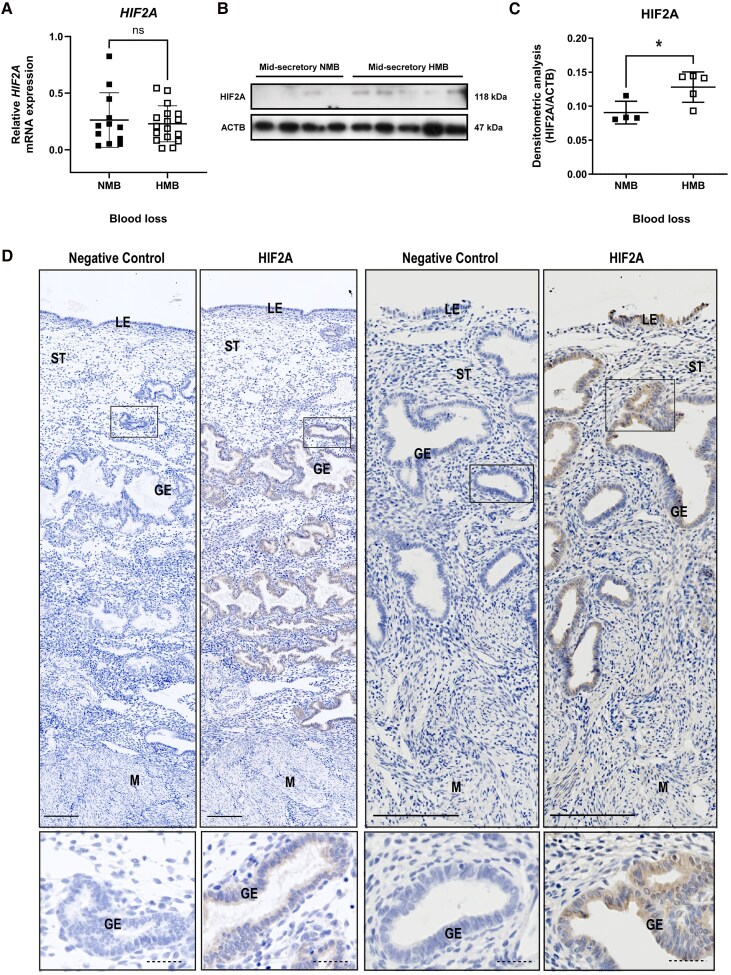
HIF2A is present in the human endometrium during the secretory phase and increased in women with HMB. (A) *HIF2A* mRNA concentration in the early-mid secretory endometrium from those with objectively measured NMB (n = 12) and HMB (n = 17). (B) HIF2A Western blot and (C) densitometry of mid-secretory phase endometrium from those with objectively measured NMB (n = 4) and HMB (n = 5). *P <.05, unpaired *t*-test. (D) HIF2A staining of human mid-secretory uterine sections comprising endometrial and myometrial layers (full-thickness sections, representative images from n = 2 individuals). Scale bar 200 μm. Magnification of the glandular epithelium. Scale bar 15 μm. Data represented as mean ± SD.

Abbreviations: ACTB, actin; GE, glandular epithelium; HIF2A, hypoxia inducible factor 2 alpha; HMB, heavy menstrual bleeding, >80 mL; LE, luminal epithelium; M, myometrium; NMB, normal menstrual bleeding, <80 mL; ST, stroma.

Since HIF1A has been associated with prompt and adequate menstrual endometrial repair (18) but the specific regulation and role of the alternative subunit HIF2A in the nonpregnant endometrium has not been fully characterized, we aimed to explore differences in endometrial HIF2A between those with objectively measured NMB (< 80 mL/cycle) and HMB ( > 80 mL/cycle). To that purpose, the early-mid secretory phase was examined, when HIF2A has been shown to be exclusively present in the endometrium (18). During this phase, *HIF2A* (*EPAS1*) mRNA concentrations were similar in the endometrium of those with NMB and HMB (Fig. 4A) However, densitometric analysis of Western blots prepared using mid-secretory endometrial nuclear protein extracts identified significantly higher HIF2A protein in samples from women with HMB when compared to those with NMB (Fig. 4B and 4C), supporting our hypothesis. To localize HIF2A protein in the endometrium, we performed immunohistochemistry on uterine samples collected at hysterectomy that comprise the mid-secretory endometrial and myometrial layers (full-thickness mid-secretory section) and revealed HIF2A was present in the glandular epithelium (Fig. 4D).

### *Hif2a^+/−^* Mice Did Not Display Compromised Decidualization or Vascular Alterations in Our Model of Simulated Menses

To ascertain the role of HIF2A in the endometrium, we first examined genetic HIF2A-deficient mice and subjected them to a simulated menses protocol (18, 31) (Fig. 1A). Whereas homozygous deletion of HIF2 (*Hif2a^−/−^*) is often lethal (34), *Hif2a^+/−^* mice remain phenotypically identical to their WT (*Hif2a*^+/+^) littermates. Only when challenged with low oxygen scenarios, impaired pulmonary vascular remodeling (24) and cardiovascular pathologies (25) arise. To confirm endometrial HIF2A deficiency, we demonstrated that uterine HIF2A protein in our mouse model of simulated menstruation was significantly decreased in *Hif2a^+/−^* compared to WT mice at the time of P4 withdrawal (t_0_) (Fig. 1B and 1C). Importantly, HIF2A partial deficiency was not compensated with HIF1A protein upregulation (Fig. 1B). Hence, the simulated model of menses in *Hif2a^+/−^* mice provides a framework in which to study the impact of decreased levels of HIF2A during simulated menstruation.

Prior to menses induction and examination of endometrial function in *Hif2a^+/−^* mice, we examined breeding as a functional measure of successful decidualization. Endometrial decidualization involves dramatic morphological and functional changes in stromal and vascular cells that occur at implantation in mice. In menstruating species, decidualization occurs spontaneously before implantation and is thought to be critical for menstruation and reproductive health (35). Therefore, we examined the breeding of *Hif2a^+/−^* and WT littermates when mated with WT fertile male mice by quantifying the total number of pups over a 3-month breeding period (Fig. 1D). During this time, all the matings had 3 litters, regardless of parental genotype. Although the number of pups between *Hif2a^+/−^* and WT females was not significantly different, a trend toward *Hif2a^+/−^* having smaller litters was observed. No deaths at birth or postnatal deaths were recorded during the duration of the experiment.

Next, we subjected WT and *Hif2a^+/−^* mice to an established model of simulated menses (18, 31) (Fig. 1A). Unlike humans, mice do not spontaneously menstruate or decidualize prior to implantation. In our mouse model of simulated menses (Fig. 1A), mice are ovariectomized before administration of sequential E2 and P4. Decidualization is induced with a transcervical injection of oil to a hormone-primed uterus, and histological features are present 96 hours later at the time of P4 withdrawal (t_0_; decidualization). Histological evidence of endometrial breakdown occurs 8 hours following withdrawal of P4 (t_8_; bleeding), and there is evidence of vaginal bleeding (18). By 24 hours following P4 withdrawal (t_24;_ repair) there is histological evidence of endometrial repair. This endometrial breakdown and repair can be graded using a previously published histological grading system [Supplementary Fig. SA (32)].

Given the detection of human endometrial HIF2A during the early/mid-secretory phase, we hypothesized that cell-cell communication in the human endometrium may result in an impact on decidualization. The percentage of mice failing to decidualize (complete absence of uterine horn thickening) in our simulated menses model was similar in *Hif2a^+/−^* and WT females (Fig. 1E). When comparing uterine mRNA concentrations of decidualization markers (23, 36) (*Bmp2*, *Hoxa10,* and the prolactin-related factors *Prl8a2*, *Prl3c1,* and *Prlr*) 4 days after the decidualization stimulus (t_0_), no significant differences were observed between *Hif2a^+/−^* and WT mice (Fig. 1F).

At decidualization, extensive remodeling of specialized human endometrial spiral arterioles takes place in preparation for implantation (37), with the vascular endothelial growth factor A (VEGF) being a key player in mediating angiogenesis and vascular development (38). Prior to P4 withdrawal (t_0_), there were no differences in *Vegf* mRNA concentrations between *Hif2a^+/−^* and WT mice (Fig. 1G). Dual staining with the endothelial marker CD31 and the mural marker alpha smooth muscle actin is considered to provide a primary assessment of vessel maturity (39, 40). In our mouse model, at t_0_, both WT and *Hif2a^+/−^* mice exhibited robust mural cell coverage around CD31^+^ endometrial endothelium (Fig. 1H).

### Partial Genetic Deficiency or Pharmacological Inhibition of HIF2A Did Not Significantly Impact the Extent of Endometrial Breakdown and Repair During Simulated Menses

To explore how HIF2A reduction affects endometrial function using the mouse model of simulated menstruation, we histologically graded endometrial breakdown/repair 8 hours (t_8_; bleeding) and 24 hours (t_24_; repair) following P4 withdrawal in WT and *Hif2a^+/−^* mice using criteria outlined in Supplementary Fig. SA (32). Assessment of endometrial breakdown/repair at t_8_ revealed that 7% of *Hif2a^+/−^* mice had already reached complete repair, a subpopulation completely absent in WT mice (Fig. 2A). However, median scores were not significantly different between *Hif2a^+/−^* and WT littermates. Histological scoring at the time of repair (t_24_) showed no significant differences between *Hif2a^+/−^* and WT littermates (Fig. 2B). Despite 41% of each experimental group achieving full repair, 20% of WT mice were still undergoing early breakdown. This early breakdown group was not present in the *Hif2a^+/−^* cohort at t_24_, suggesting a nonsignificant trend of enhanced repair with lower HIF2A (Fig. 2B), consistent with our findings in women (Fig. 4B and 4C). In order to measure MBL, mice were fitted with cotton pellets (tampons) and the collected blood was quantified using an adapted version of the alkaline hematin method (29), a standard measurement procedure for MBL quantification in women. During active bleeding (t_8_), there was a nonsignificant trend of decreased MBL in *Hif2a^+/−^* compared to WT (Fig. 2C). Due to high rates of tampon loss, it was not possible to quantify MBL at t_24_.

We also investigated how HIF2A deficiency affected menstrual uterine tissue hypoxia. We have shown that endometrial hypoxia is required for normal repair of the denuded endometrial surface at menstruation to limit MBL (18). Pimonidazole positive staining is an indicator of tissue hypoxia, forming detectable protein adducts when the oxygen levels are lower than 10 mmHg (16). Consistent with previous findings (18), endometrial hypoxia was present during active bleeding (t_8_) in our *Hif2a^+/−^* mouse model of menses (Fig. 2D). The extent of the hypoxic area did not significantly vary between *Hif2a^+/−^* and WT mice (Fig. 2E). When examining a panel of hypoxia/HIF downstream targets (*Slc2a1, Cxcr4, Ccnd1, Vegf*) at t_8_, most were not altered with the exception of significantly lower glucose transporter protein type 1 (*Slc2a1*) that was observed in *Hif2a^+/−^* mice compared to WT (Fig. 2F). At t_24_, the hypoxia-regulated mRNAs C-X-C chemokine receptor type 4 (*Cxcr4*) and cyclin D1 (*Ccnd1*) were significantly decreased in *Hif2a^+/−^* mice compared to their WT counterparts (Fig. 2G).

The *Hif2a^+/−^* genetic model provides a consistent constitutive decrease of HIF2A protein levels across the simulated menstrual cycle. As our findings in human endometrium revealed HIF2A protein was exclusively present in the secretory phase, we next pharmacologically inhibited HIF2A on C57BL/6JOlaHsd mice at the time of decidualization. This timepoint is equivalent to the secretory phase in the human menstrual cycle (18). To achieve temporal inhibition, the HIF2A selective inhibitor PT2385 was orally administered in 4 doses: 7 hours prior to the decidualization stimulus and 5 hours, 17 hours, and 29 hours postdecidualization (Fig. 2H). Endometrial tissue analysis 4 hours after the second dose administration showed significantly lower mRNA concentrations of HIF binding targets (Fig. 2I), confirming inhibition of HIF2 in uterine tissue. However, mice examined at t_24_, after administration of all 4 doses of PT2385, did not show any significant impact on endometrial breakdown and repair grades compared to the control group (Fig. 2J). At this timepoint, a significant decrease in *Ccnd1* was detected in PT2385-treated mice compared to controls (Fig. 2K).

Taken together, partial genetic or pharmacological inhibition of HIF2A does not appear to significantly alter the timing of endometrial repair or modify uterine blood loss in our mouse model. However, we observed more partially HIF2 deficient animals achieving complete endometrial repair at t_24_ and a corresponding nonsignificant decrease in MBL at t_8_.

### Pharmacological Stabilization of HIF2A in Mice at the Time of Decidualization Had No Significant Impact on Tissue Endometrial Hypoxia, MBL and Endometrial Repair During Simulated Menses

To investigate the impact of the increased HIF2A during decidualization that we observed in women with HMB, HIF2A was pharmacologically stabilized in our mouse model of simulated menses. Since specific HIF2 stabilizers are not yet available, the pan-HIF inhibitor DMOG was used. DMOG is capable of stabilizing both HIF1 and HIF2 in normoxic conditions (41). To selectively increase HIF2 during decidualization, *Hif1a^+/−^* mice were treated with 2 doses of DMOG 24 hours apart 3 to 4 days prior to P4 withdrawal (t_0_). Through this approach, pharmacological inhibition will primarily target HIF2A as *Hif1a^+/−^* mice are unable to mount an appropriate HIF1A response when challenged with hypoxia (Fig. 3A and 3B). As expected, 8 hours after the second dose, we observed increased protein levels of HIF2 in DMOG-treated vs vehicle-treated mice but did not see a simultaneous increase in HIF1A protein (Fig. 3C).

Pharmacological stabilization of HIF2A did not affect rates of decidualization in our mouse model of menses, with similar rates in vehicle- and DMOG-treated mice (Fig. 3D). When examining mRNA concentrations of markers of decidualization (Fig. 3E), vascular remodeling (Fig. 3F) and vessel maturity [Supplementary Fig. SB (32)] at t_0_, no differences were observed between the 2 experimental groups.

During active uterine bleeding (t_8_), histological assessment of breakdown and repair showed no significant difference between vehicle- and DMOG-treated mice (Fig. 3G). However, 55% of DMOG-treated mice were undergoing early breakdown, compared to 23% in the vehicle group. Similarly, only 11% of DMOG-treated mice had reached early repair during active uterine bleeding (t_8_), compared to 30% of vehicle-treated mice. This is consistent with a slower progression through the breakdown to early repair phases with HIF2A stabilization. At the time of repair (t_24_), there were no significant differences in endometrial repair in vehicle vs DMOG-treated mice (Fig. 3H) with ∼50% of both groups reaching full repair, suggesting that an increase in HIF2A does not result in prolonged menstrual bleeding or a phenotype of HMB when equated to humans.

Pharmacological stabilization of HIF2A with DMOG did not affect the amount of uterine blood loss (Fig. 3I) or the extent of endometrial hypoxia (Fig. 3J and 3K) as assessed by pimonidazole staining compared to vehicle-treated mice. However, examination of general HIF-regulated genes during endometrial breakdown (t_8_) revealed a statistically significant downregulation of *Cxcr4* as well as upregulation of *Vegf* in DMOG-treated mice compared to controls (Fig. 3L). *Vegf* was also higher in DMOG-treated mice during the initiation of repair (t_24_) phase (Fig. 3M).

We conclude that, in our mouse model of simulated menses, pharmacological stabilization of HIF2A at the time of decidualization had no statistically significant impact on endometrial hypoxia, MBL, or endometrial repair. However, there were nonsignificant trends of lower histological breakdown/repair scores that are consistent with our findings of increased HIF2A protein in the secretory endometrium of women experiencing HMB.

## Discussion

The development of new, effective non-hormonal treatments for the symptoms of HMB has been hindered due to the limited understanding of the cellular and molecular processes underpinning excessive endometrial bleeding (42). Here we reveal that women with HMB exhibited increased endometrial HIF2A protein during the P4-dominant mid-secretory phase of the menstrual cycle compared to those with NMB. HIF2A partial genetic deficiency in our mouse model of simulated menses showed nonsignificant trends in accelerated endometrial repair and reduced MBL. Pharmacological stabilization of HIF2A in mice at the time of decidualization, when P4 levels are high, did not yield statistically significant effects on MBL or endometrial repair but revealed some nonsignificant delays in endometrial breakdown/repair that are consistent with our findings of increased endometrial HIF2 protein in women with objectively measured HMB (> 80 mL/cycle). These data imply that aberrant HIF2A levels alone are insufficient to result in the phenotype of HMB. However, HIF2A may be one of many contributors that optimize endometrial function for menstruation, such as HIF1A, NFκB, and glucocorticoids (9).

Herein we reveal that women with objectively quantified HMB displayed increased levels of endometrial HIF2A during the mid-secretory phase compared to those with NMB, despite small numbers. Temporal changes in endometrial HIF2A across the menstrual cycle have been previously documented (18), with exclusive presence during the early-mid secretory phase. Although hypoxia is the canonical trigger for HIF pathway activation (19), the lack of hypoxia observed during the secretory phase of the cycle (17) suggests alternative HIF modulation (43). Previous studies have shown that E2 administration induces HIF2A, both in P4-primed stromal cells in vitro (44) and in the uterus of ovariectomized mice (45). Whether other mediators present during the secretory phase are responsible for endometrial HIF2 upregulation, such as reactive oxygen species (46) or NFκB modulation (47), remains to be determined.

In this manuscript we have focused on the role of HIF2A in the nonpregnant endometrium, taking a loss of function and gain of function approach in the mouse model of simulated menstruation. Using a constitutive loss of function approach, we subjected HIF2A heterozygote mice to our mouse model of simulated menstruation. Assessment of repair at the time of active bleeding (t_8_) and early repair (t_24_) in this partial deficiency of the HIF2A system revealed mRNA downregulation of hypoxia downstream binding targets important in endometrial conditioning (*Slc2a1*) (48), cell proliferation (*Ccnd1*), and homing (*Cxcr4*) (49). Histologic assessment also showed trends of enhanced repair at both timepoints that, despite not reaching statistical significance, may have clinical relevance. This is supported by the nonsignificant decrease in MBL in *Hif2a^+/−^* mice. Surprisingly, pharmacologically restricting HIF2A inhibition around the time of decidualization as opposed to a consistent but partial genetic deficiency (*Hif2a^+/−^*) during the menstrual cycle did not generate a phenotype of enhanced repair during active bleeding or early repair. This may be due to the lack of a cumulative effect of decreased HIF2A over time, with any effects of decreased HIF2 on endometrial function potentially only being revealed after multiple cycles. Alternatively, this may be due to the limitations of the mouse model of simulated menses. This model has proven to be extremely useful for delineating the cellular and molecular mechanisms that underpin menstruation (10, 50). However, the histological assessment of macroscopic breakdown and repair in this model may lack sensitivity to detect small changes. While the detection of large changes is more likely to have clinical significance and result in quantifiable phenotypic effects, more subtle changes that fine-tune endometrial function during breakdown and repair will remain undetected or fail to reach statistical significance. MBL analysis in this model also presents challenges. We acknowledge that retrieval of tampons both at the time of active endometrial breakdown (t_8_) and during early repair (t_24_) would provide an ideal assessment of MBL. However, t_24_ MBL analysis was technically not possible due to the presence of shed tissue embedded in the tampon as well as gnawing of the vaginal suture that keeps the tampons in place by mice after 8 hours of use. Our animal ethics prevented a further nonsurgical procedure to change the tampon after 8 hours. This limited our assessment of MBL to the first 8 hours following P4 withdrawal, equating to active endometrial breakdown in humans. However, as HIF2A is exclusively present in the endometrium premenstrually, we anticipate that blood loss in this first 8 hours in our mice will be reflective of any functional effects of HIF2A, eg, any potential impact on vasoconstriction and subsequent MBL (9, 51).

Focusing on the gain of function strategy, stabilization of HIF2A to gain mechanistic insight on the phenotype of HMB is challenging due to a lack of specific pharmacologic HIF2A stabilizers. When selectively increasing HIF2 by treating *Hif1a^+/−^* mice with the general HIF stabilizer DMOG (Fig. 3A and 3B), treated mice displayed a trend of delayed endometrial repair 8 hours after P4 withdrawal (t_8_) that resolved at the time of early repair (t_24_). DMOG administration is known to activate VEGF-dependent repair mechanisms (52, 53), consistent with the sustained VEGF mRNA upregulation we observed both at t_8_ and t_24_ timepoints but not at t_0_. Of note, *Hif1a^+/−^* mice already display delayed endometrial repair (18), effectively serving as a model for HMB without further pharmacological manipulation of the HIF pathway. Thus, partial deficiency of HIF1A may mask subtle effects of increased HIF2. However, this method does represent our findings in women with HMB, who have demonstrated reduced HIF1A at menstruation (18) and the increased HIF2 identified premenstrually herein. Our findings of reversion to optimal repair with no significant evidence of prolonged MBL suggests that the increased protein levels of HIF2A may contribute to but are not sufficient for the clinical manifestation of HMB. Interestingly, the in vitro generation of a gain of function mutation in smooth muscle cells-derived induced pluripotent stem cells (iPSCs) compromised their vascular mechanics (54). This same mutation in transgenic mice (*HIF2a*^G536W/+^ mice) revealed pulmonary hypertension due to disorganised actin architecture in the smooth muscle cells isolated from their pulmonary arteries (54). Whether this HIF2A gain of function mouse model has endometrial impact or replicates the phenotype of HMB when subjected to simulated menses remains unknown.

The implication of HIF2A in endometrial receptivity and early pregnancy establishment has been robustly supported in the past decade. Mice with uterine deletion of HIF2A exhibit normal embryo apposition; however, preservation of the intact luminal epithelium surrounding the embryo results in failed embryo invasion, impaired vascular network development, and a phenotype of infertility (23, 44). Herein, our *Hif2a^+/−^* females were fertile and showed a nonsignificant trend toward having smaller litters than WT females when mated with WT males. Similarly, when subjected to the mouse model of simulated menses, *Hif2a^+/−^* mice demonstrated similar rates of successful decidualization and mRNA concentrations of markers of decidualization *(Bmp2, Hoxa10, Prl8a2, Prl3c1, Prlr)* at t_0_ when compared to WT littermates. Furthermore, when HIF2A was pharmacologically stabilized at decidualization, again no significant changes in these markers were observed. Therefore, these data suggest that decidualization is only minimally affected by HIF2A, if at all, in the nonpregnant endometrium.

Vascular remodeling and maturation of specialized endometrial blood vessels is crucial for implantation and future placentation (37) but may also fine-tune menstruation through endometrial hemostasis (55). The role of HIF2A in systemic angiogenesis and vasculogenesis is well characterized (56, 57), and uterine deletion of *HIF2A* provides evidence of its contribution to successful endometrial perfusion during implantation (44), but its involvement in endometrial vascular conditioning prior to menstruation remains unclear. In our mouse model of menses, *Hif2a^+/−^* showed consistent coverage and staining of mural cells surrounding CD31^+^ endothelial cells when decidualized (t_0,_ P4 levels high), resembling similar endometrial vascular maturity to WT littermates. This was also the case when comparing the vasculature of DMOG-treated *Hif1a^+/−^* mice to vehicle controls (t_0_ timepoint) Previous assessment of human mid-secretory endometrial vasculature in those with HMB revealed evidence of vascular immaturity (37). We did not observe this in our mouse model of decreased HIF2A, although we acknowledge the differences in endometrial vasculature between humans and mice (58, 59). Our studies also revealed that the extent of hypoxia was not affected by the genetic or pharmacological manipulation of HIF2A, suggesting that the endometrial hypoxic insult required to stabilize HIF1A during menstruation is unlikely to be dependent on HIF2-mediated changes in vasoconstriction. Although endometrial vasculature and decidualization were not significantly altered by manipulation of HIF2 prior to P4 withdrawal in our mouse model, nonsignificant changes in endometrial function premenstrually may result in clinically relevant effects on vasoconstriction and endometrial hypoxia leading to increased blood loss at menstruation. Although these changes were not detected in our simulated mouse model, it remains possible that the increased HIF2A detected premenstrually in the endometrium from women with HMB may affect subsequent MBL.

In conclusion, we detail the presence and role of HIF2A in the nonpregnant endometrium revealing increased secretory endometrial HIF2A in women with objective HMB. While our findings in the mouse model of simulated menstruation suggest that HIF2A inhibition is unlikely to provide a stand-alone therapeutic target for those with HMB, limiting HIF2A during the secretory phase may fine-tune endometrial function at menstruation.

## Data Availability

Upon reasonable request, all relevant data are available from the authors.
