# Correction to: “Limiting Premenstrual Endometrial Hypoxia Inducible Factor 2 Alpha May Fine-Tune Endometrial Function at Menstruation”

**DOI:** 10.1210/clinem/dgaf493

**Published:** 2025-09-05

In the above-named article by Martínez-Aguilar R, Rowley BM, Walker C, Critchley HOD, Carmeliet P, and Maybin JA (*J Clin Endocrinol Metab*. 2025; 110(4), 1135-1147; doi: 10.1210/clinem/dgae630) there was an error in the Methods section.

In the originally published article, in the Methods section, under the “Human Tissue Collection” subsection, the original sentence stated, “Written informed consent was obtained from participants and ethical approval granted from Lothian Research Ethics Committee (REC 08/S1103/38, 10/S1402/59).” In the corrected article, the ethics numbers were updated and the sentence now reads, “Written informed consent was obtained from participants and ethical approval granted from Lothian Research Ethics Committee (REC 07/S1103/29, 20/ES/0119).”

The article has been corrected online.

doi: 10.1210/clinem/dgae630

